# Hope Hospital, Salford

**Published:** 1910-11-12

**Authors:** 


### HOPE HOSPITAL, SALFORD.

### THE APPOINTMENT OF MEDICAL SUPERINTENDENT.

Some confusion has arisen in the public mind—and it is not credible that the majority of the Guardians of the Salford Union themselves understand the position—with regard to the point at issue between the Local Government Board and the Guardians. The Guardians would never have kept their hospital before the public in an unenviable light for some months past, if they discharged their duties judicially and in a businesslike way by adhering to the conditions laid down in their own advertisement inviting candidates for the post of medical superintendent. This advertisement stated that applicants must be fully qualified under the Medical Acts and also have had previous experience in a Poor-Law or general infirmary. In response to that advertisement a number of excellently qualified members of the profession, who had the required experience and were highly qualified, applied for the post. Instead of electing a qualified applicant, the majority of the Board gave their votes for a gentleman who was clearly disqualified for the appointment, seeing that he had not had the previous administrative experience in a Poor-Law or general infirmary, which was laid down as a condition precedent in the advertisement. The more intelligent of the Guardians must have known that the selected candidate had not had the experience required since he took his degree, and that he had been for fourteen years in general practice. By an overwhelming majority the Guardians, however, ignored the conditions they had laid down and proceeded to elect the candidate of their choice, though disqualified. When the appointment went forward for confirmation by the Local Government Board Mr. John Burns and his advisers at once fastened on the weak point, and endeavoured to get the Guardians to put themselves in order, and to do their duty. Correspondence and an interview took place between the Salford Guardians and the Local Government Board, and in a letter dated September 14 the Board refused to approve the appointment irregularly made, and the assistant secretary set forth the facts clearly as follows:—

I am directed by the Local Government Board to advert to your letter of the 3rd instant, with reference to the proposed appointment of Mr. H. Yearnshaw as medical superintendent of the infirmary of the Salford Union.

The Board have given careful consideration to this matter, and they regard it as essential that the medical superintendent of so important an institution should have had a wide and recent experience of hospital administration. They gather from the papers forwarded that Mr. Yearnshaw has been in general practice for the last fourteen years, and that the infirmary experience to which the testimonials refer took place before that time, and was mainly, if not entirely, before he became qualified. This being so, they do not regard this experience, or his experience as deputy to the medical officer of the workhouse, as sufficient to justify his selection for the post of medical superintendent. They are, therefore, not prepared to assent to his appointment, and they must request the Guardians to proceed to a fresh appointment.

Instead of accepting the position and recognising the important public interests involved, the leaders of the majority of the Guardians refused to recognise that they were at fault from beginning to end, and tried to cast the blame upon the Local Government Board. This latter Board remained firm, however, and in the result the appointment of medical superintendent has been re-advertised, and the Guardians will proceed to select one of the new candidates at their meeting which will, we understand, be held on or about the 12th instant.

The action of the Local Government Board in this matter reveals once more the thoroughness of Mr. John Burns, and the deep interest which he takes in the welfare of the poor people who seek treatment in Poor-Law infirmaries. We hope that the Salford Guardians will this time be careful to select the best qualified candidate who presents himself, and who may be possessed of the widest practical experience in the administrative work of a Poor-Law or general infirmary. Such a step is essential if the good work of improvement and development at the Hope Hospital, which has been successfully initiated by the retiring superintendent, Dr. Hindshaw, is to continue. That work has set up a better standard and reduced the internal administration to good order, so that if a competent member of the profession, with administrative gifts and sound practical experience, be appointed, the much-needed improvements already initiated should continue and soon become permanent features of excellence.

No amount of rhetoric can conceal the plain fact that a majority of the Guardians at the late election ignored the conditions laid down by their own advertisement, and proceeded to elect a gentleman to this important post who, as the Local Government Board reminded them, had been in general practice for fourteen years, whilst the infirmary experience to which his testimonials referred took place mainly, if not entirely, before he became a qualified member of the medical profession. The ratepayers of Salford should grasp these facts, and if they desire to have Hope Hospital efficiently administered in future they will take an opportunity of marking their disapproval of the conduct of certain Guardians which was as unbusinesslike as it was indefensible.

